# Controlling the Last Known Cluster of Ebola Virus Disease — Liberia, January–February 2015

**Published:** 2015-05-15

**Authors:** Tolbert Nyenswah, Mosoka Fallah, Sonpon Sieh, Karsor Kollie, Moses Badio, Alvin Gray, Priscilla Dilah, Marnijina Shannon, Stanley Duwor, Chikwe Ihekweazu, Thierry Cordier-Lasalle, Shivam A. Shinde, Esther Hamblion, Gloria Davies-Wayne, Murugan Ratnesh, Christopher Dye, Jonathan S. Yoder, Peter McElroy, Brooke Hoots, Athalia Christie, John Vertefeuille, Sonja J. Olsen, A. Scott Laney, Joyce J. Neal, Thomas R. Navin, Stewart Coulter, Paran Pordell, Terrence Lo, Carl Kinkade, Frank Mahoney

**Affiliations:** 1 Ministry of Health and Social Welfare, Liberia; 2 World Health Organization; 3 CDC

As one of the three West African countries highly affected by the 2014–2015 Ebola virus disease (Ebola) epidemic, Liberia reported approximately 10,000 cases (1). The Ebola epidemic in Liberia was marked by intense urban transmission, multiple community outbreaks with source cases occurring in patients coming from the urban areas, and outbreaks in health care facilities (HCFs) (2,3). This report, based on data from routine case investigations and contact tracing, describes efforts to stop the last known chain of Ebola transmission in Liberia. The index patient became ill on December 29, 2014, and the last of 21 associated cases was in a patient admitted into an Ebola treatment unit (ETU) on February 18, 2015. The chain of transmission was stopped because of early detection of new cases; identification, monitoring, and support of contacts in acceptable settings; effective triage within the health care system; and rapid isolation of symptomatic contacts. In addition, a “sector” approach, which divided Montserrado County into geographic units, facilitated the ability of response teams to rapidly respond to community needs. In the final stages of the outbreak, intensive coordination among partners and engagement of community leaders were needed to stop transmission in densely populated Montserrado County. A companion report describes the efforts to enhance infection prevention and control efforts in HCFs (4). After February 19, no additional clusters of Ebola cases have been detected in Liberia.* On May 9, the World Health Organization declared the end of the Ebola outbreak in Liberia.

## Evolution of the Cluster

The index patient in this cluster was a woman aged 50 years who became ill on December 29, 2014, in a community near St. Paul River Bridge in Montserrado County (Monrovia). After seeking care from an herbalist in her community, the patient presented to an HCF on January 4 with high fever, red eyes, and cough. Ebola was suspected, but she refused referral to an ETU and was sent home with antibiotics and antipyretics. On January 5, she was admitted to an ETU and died later that day. A postmortem swab of oral fluids tested positive for Ebola virus by polymerase chain reaction. Her family reported no known contact with other Ebola patients, although other Ebola cases had been reported in the same neighborhood. In addition, before her illness, the woman had traveled to Grand Cape Mount County, where Ebola virus transmission was ongoing.

**FIGURE 1:**
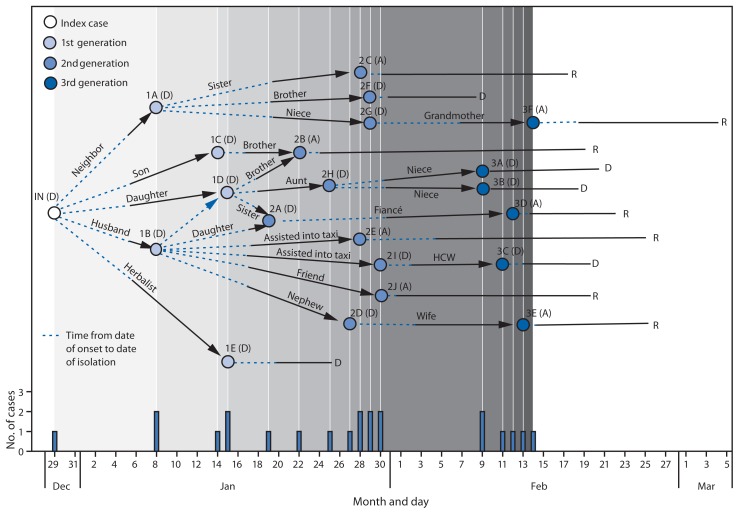
Transmission diagram for the last known cluster of Ebola virus disease cases (N = 22) — Liberia, December 29, 2014–March 5, 2015* **Abbreviations:** D = dead; A = alive; R = recovered. * In this transmission network diagram, date of onset of Ebola symptoms of confirmed cases (dot) is followed by a period of infectiousness (dotted line); time from date or isolation or safe burial to onset of the next generation case (black arrow); and time from date of isolation or safe burial to final disposition (solid black line). Dot color represents generation. Cases are identified by a two character abbreviation: generation number and sequential lettering based on onset date. Survival status is indicated after each case abbreviation.

**FIGURE 2:**
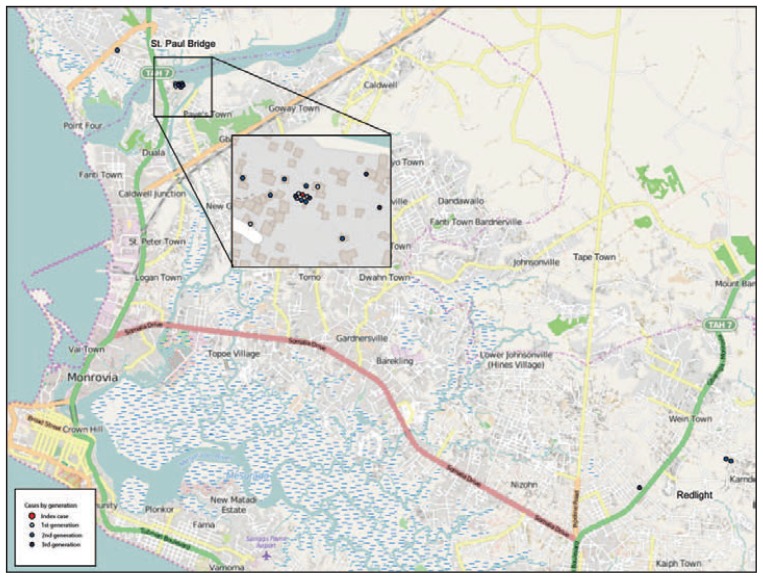
Ebola virus disease (Ebola) cases (N = 21) in the last known cluster of Ebola, by location and transmission generation — Montserrado County,* Liberia, January–February 2015 * N = 21 for Montserrado County; one other case in this cluster of 22 cases occurred in Margibi County.

Over the following 7 weeks, 21 additional persons with laboratory-confirmed Ebola were linked to this case: 11 family members, six neighbors, two community members, one health care worker, and an herbalist (Figure 1). These cases occurred in three generations, all epidemiologically linked to the index case. The time interval from onset of illness to admission to an ETU decreased with each generation of cases. Twenty patients (including the index patient) received treatment at an ETU, including 13 patients who died. The two associated Ebola-infected persons who did not seek care in an ETU died in the community. Five first-generation patients were admitted to an ETU on average 6.0 days (range = 2–11 days) after illness onset. Ten second-generation patients averaged 4.7 days (range = 1–11 days) from symptom onset to ETU admission or death in the community. The six third-generation patients averaged 1.5 days (range = 0–4 days) from symptom onset to ETU admission (Table). The case-fatality rates among the successive generations were 100%, 60%, and 50%, respectively. Probable transmission for 18 of the cases (86%) occurred within 1 kilometer of St. Paul River Bridge in Montserrado County, whereas transmission for three cases occurred near Red Light, 15 kilometers southeast of St. Paul River Bridge (Figure 2).

Five patients worked in an HCF, three as cleaners (1A, 2C, and 3D) and two as health care providers (3A and 3C). However, the cleaners and one health care provider (3A) had significant household exposures with persons with confirmed Ebola that could account for their infection (Figure 1). One patient (1B) traveled to Red Light while symptomatic, became incapacitated in the community, and exposed two persons (2E and 2I) who assisted him into a taxi. One of these men later exposed patient 3C, a health care provider working in Red Light.

According to information provided by patients or their family during case investigations, several symptomatic patients sought care in counties outside of Montserrado to conceal their illness or obtain more affordable medical care. Patient 2A traveled from Montserrado to Bomi County to seek care at an ETU; 2G traveled to Bomi County to access an affordable appendectomy, but was turned back at a county checkpoint; 2H traveled from Montserrado to Lofa County and was transported by ambulance to an ETU in Bomi; and 2D, to avoid detection, traveled to Margibi County under a different name, sought care twice from a non-ETU HCF, and died there in the community (Figure 1). His wife (3E) resided in Margibi County and became infected while caring for him. At least eight patients sought care at non-ETU HCFs before their Ebola diagnosis in nine facilities in Montserrado County and one in Margibi County, exposing a total of 166 health care workers (4).

In several instances, challenges with HCF triage contributed to missing patients with suspect or probable Ebola. One patient (1A) tested positive for malaria and was sent home from an HCF. One initially afebrile patient (2G), with clinical symptoms consistent with appendicitis or pelvic inflammatory disease, received care at two clinics and was hospitalized at a third facility for 7 days before being transferred to an ETU. A symptomatic, high-risk contact (3C) under daily monitoring, presented for care at an ETU but was sent home despite a history of exposure to body fluids of a confirmed Ebola patient because his temperature was <100.4°F (<38.0°C). Two days later, he presented with symptoms at the non-ETU HCF where he worked and was sent to an ETU, where Ebola was confirmed.

Contact tracing identified 745 contacts for this cluster over the 6-week period, including the 166 health care workers from 10 HCFs (4). During the response to this cluster, considerable efforts were made to address the needs of high-risk contacts (e.g., those with documented exposure to body fluids of persons with confirmed Ebola). In some instances, contacts agreed to home-based quarantine, and groups of contacts agreed to facility-based observation (i.e., direct daily symptom and temperature monitoring in an HCF), where they could be immediately isolated if symptoms developed, without risk of community transmission. Incomplete contact tracing contributed to the persistence of this cluster; only 15 (68%) of the cases were in persons listed as known contacts; 60% of first- and second-generation and 100% of third-generation cases were in persons who were known contacts (Table). Several patients in the cluster denied Ebola symptoms or exposure to persons with confirmed Ebola when seeking care, reportedly because of fear of community stigma and apprehension of ETUs. At least one child (1D) was hidden from contact tracers when they visited. Persons who initially presented to non-ETU HCFs were less likely to be listed as contacts; two (25%) of eight persons who initially presented to non-ETUs were known contacts, compared with 13 (93%) of 14 who first presented for care at an ETU. Although guidance called for immediate isolation of symptomatic contacts, nine (75%) known contacts were isolated ≥2 days after symptom onset. The last confirmed case in this cluster (3F) was in a person admitted to an ETU on February 18 and discharged on March 5. The last cluster-associated contacts who did not become ill exited monitoring on March 11.

What is already known on this topic?

During the initial phases of the 2014–2015 Ebola virus disease (Ebola) epidemic in Liberia, there was intense urban transmission, multiple community outbreaks with source cases occurring in patients coming from the urban areas, and outbreaks in health care facilities.

What is added by this report?

The last cluster of Ebola in Liberia included 22 cases, with three generations of transmission. Through enhanced control efforts, patients in successive generations were admitted to Ebola treatment units more quickly, mortality decreased, and community transmission was interrupted.

What are the implications for public health practice?

The last chain of transmission was controlled because of successful implementation of known strategies to control Ebola, including early detection of new cases; identification, monitoring, and support of contacts in acceptable settings; effective triage within the health care system; and rapid isolation of symptomatic contacts.

### Discussion

This network of Ebola transmission in Liberia illustrated numerous challenges that persisted throughout the epidemic: fear of stigmatization in the community, delays in seeking treatment, inadequate triage in HCFs, lack of recognition of Ebola cases, and incomplete identification and follow-up of some contacts. The motivations for denying Ebola symptoms and resisting treatment are complex, but include stigma, fear, and denial related to possible Ebola infection, mistrust of ETUs, and low medical literacy. Despite the widespread availability of ETUs in Montserrado County, some persons opted for care at distant ETUs or care in non-ETU settings, where, consequently, large numbers of health care workers were exposed. Delayed treatment might have contributed to worse outcomes in the first two transmission generations compared with the last generation, when patients sought care more promptly. Triage systems did not fully prevent Ebola patients from being admitted to HCFs rather than ETUs. Despite these challenges, the last cluster of Ebola in Liberia was controlled because of successful implementation of known effective Ebola control strategies, including early detection of new cases; identification, daily monitoring, and support of contacts in acceptable settings; effective triage within the health care system; and rapid isolation of symptomatic contacts (2,3).

To improve case investigations and contact tracing, Montserrado County had coincidentally decentralized management of outbreak activities in the four geographic sectors. This decentralized, “sector” approach might have reduced the risk for community transmission. Each geographic sector had multidisciplinary teams led by coordinators located in each sector to manage and coordinate outbreak response activities at the sector, zone, and block level. Sector teams were empowered to make decisions related to control activities locally, and this enabled flexible adaptation of accepted outbreak control principles to fit local circumstances. Strategies included the use of home-based and community quarantine and facility-based observation, with provision of basic needs and psychosocial support, active case-finding, and outreach to religious and community leaders to allay the fears of affected households and community members. Although decentralization of sector management presented initial communication and coordination challenges, the enhanced sector-based efforts resulted in more complete contact tracing, more prompt isolation of symptomatic patients in the second and third generations of transmission, increased survival, and reduced transmission in the community.

As the threat of Ebola wanes, much needed non-Ebola health services are resuming in Liberia. However, comprehensive triage for Ebola (3) and appropriate personal protective equipment are crucial but cannot completely eliminate risk for Ebola transmission at HCFs. At least eight cases in the cluster described in this report were in patients who sought care at non-ETU HCFs; six (75%) of these were not listed as contacts, highlighting the critical importance of comprehensive contact tracing. These eight patients were treated by HCFs despite the universal requirement of triage. At least four patients in this cluster did not have fever when presenting for care; some HCFs and contact tracers used lack of fever as a *de facto* indicator to rule out Ebola (i.e., rather than completing a comprehensive triage), highlighting the limitations of temperature-based triage. Conversely, many non-Ebola patients had illnesses that met the case definition but could not be tested without transfer to an ETU, where care for their non-Ebola medical conditions would not be offered. Despite these challenges, only one of the exposed health care workers in this cluster became infected with Ebola, and no additional transmission occurred in HCFs, possibly because of timely, targeted infection prevention and control training and provision of personal protective equipment (4). Additionally, the most recent Ebola patient was appropriately triaged to an ETU when she presented to a non-ETU HCF (5).

In contrast to earlier in the Ebola epidemic, sector-based intensified contact tracing and in-depth case investigation, widespread infection prevention and control efforts (3), and coordination of case investigation and contact tracing activities between Montserrado and other counties (6) were key to stopping this final chain of Ebola transmission. The risk for re-introduction of Ebola into Liberia will remain high as long as transmission continues in the region. National efforts to strengthen surveillance, alert and response, border screening, and triage and infection prevention and control in HCFs are high-priority activities in the government of Liberia’s recovery plan.

## Figures and Tables

**TABLE:** Characteristics of patients with Ebola virus disease (Ebola) in the last known cluster of Ebola (N = 22*), by transmission generation — Liberia, January–February 2015

Characteristic	Transmission generation			

	Total (N = 22)*	1st (n = 5)	2nd (n = 10)	3rd (n = 6)
**Average age (yrs) (range)**	**36 (10–60)**	32 (10–60)	34 (13–55)	41 (24–58)
**Average no. of symptomatic days in the community (range)**	**4.2 (0–11)**	6 (2–11)	4.7 (1–11)	1.5 (0–4)
**Female**	**12**	2	5	4
**Survived**	**7**	0	4	3
**Transmission location**				
Montserrado County, Sector 2	**18**	5	8	4
Montserrado County, Sector 4	**3**	0	2	1
Margibi County	**1**	0	0	1
**Initially listed as contact**	**15**	3	6	6
**Visited non-ETU while symptomatic**	**8**	2	4	1

**Abbreviation:** ETU = Ebola treatment unit.

* Includes index patient.

## Footnotes

* Another single case occurred in a person who received a diagnosis of Ebola on March 20, 2015, and was not connected to this cluster (5).
